# Stimulation of the Midbrain Periaqueductal Gray Modulates Preinspiratory Neurons in the Ventrolateral Medulla in the Rat In Vivo

**DOI:** 10.1002/cne.23334

**Published:** 2013-04-29

**Authors:** Hari H Subramanian, Gert Holstege

**Affiliations:** 1 The University of Queensland Centre for Clinical ResearchHerston, Queensland, 4029, Australia; 2 Discipline of Biomedical Science, The University of SydneySydney, New South Wales, 2006, Australia; 3 Center for Uroneurology, University of Groningen, University Medical Center Groningen9713 GZ Groningen, The Netherlands

**Keywords:** periaqueductal gray, pre-Bötzinger, pre-I neuron, inspiration, respiration, emotional behavior

## Abstract

The midbrain periaqueductal gray (PAG) is involved in many basic survival behaviors that affect respiration. We hypothesized that the PAG promotes these behaviors by changing the firing of preinspiratory (pre-I) neurons in the pre-Bötzinger complex, a cell group thought to be important in generating respiratory rhythm. We tested this hypothesis by recording single unit activity of pre-Bötzinger pre-I neurons during stimulation in different parts of the PAG. Stimulation in the dorsal PAG increased the firing of pre-I neurons, resulting in tachypnea. Stimulation in the medial part of the lateral PAG converted the pre-I neurons into inspiratory phase-spanning cells, resulting in inspiratory apneusis. Stimulation in the lateral part of the lateral PAG generated an early onset of the pre-I neuronal discharge, which continued throughout the inspiratory phase, while at the same time attenuating diaphragm contraction. Stimulation in the ventral part of the lateral PAG induced tachypnea but inhibited pre-I cell firing, whereas stimulation in the ventrolateral PAG inhibited not only pre-I cells but also the diaphragm, leading to apnea. These findings show that PAG stimulation changes the activity of the pre-Bötzinger pre-I neurons. These changes are in line with the different behaviors generated by the PAG, such as the dorsal PAG generating avoidance behavior, the lateral PAG generating fight and flight, and the ventrolateral PAG generating freezing and immobility. J. Comp. Neurol. 521: 3083–3098, 2013. © 2013 Wiley Periodicals, Inc.

The midbrain periaqueductal gray (PAG) is involved in many different behaviors Holstege, 1991, 1992, such as flight and aggression (Bandler and Carrive, 1988); pain and anxiety (Lovick, 2000); fear, freezing, and immobility (Zhang et al., 1990; Carrive, 1993); micturition (Holstege, 2010; Stone et al., 2011); and vocalization (Holstege, 1989; Zhang et al., 1994; Subramanian et al., 2008). Many of these behaviors affect breathing. For example, if an individual finds himself in a dangerous situation, the frequency as well as the depth of breathing has to be adjusted in order to fight or to escape the threat (Subramanian et al., 2008; Subramanian and Holstege, 2010). Expression of emotions through vocalization in animals and speech in humans requires changes in the expiratory effort (Subramanian et al., 2008). Vocalization, for example, not only requires expiratory modulation but also involves changes in inspiratory rhythm (Subramanian et al., 2008). The basic or eupneic inspiratory rhythm is thought to be generated and maintained by the preinspiratory (pre-I) neurons located in the pre-Bötzinger complex (pre-BötC) region of the ventrolateral medulla (Smith et al., 1991; Johnson et al., 1994; Paton, 1996; Ramirez and Richter, 1996; Koshiya and Smith, 1999; Rekling et al., 2000; Richter and Spyer, 2001; Gray et al., 2001; Feldman and Del Negro, 2006; Smith et al., 2009; Feldman et al., 2012). These cells not only exhibit rhythmic bursting in vitro but also in vivo during the expiration–inspiration transition (Schwarzacher et al., 1995; Guyenet and Wang, 2001), which suggests that they form the inspiratory ON switch. We hypothesize that the PAG changes the firing of the pre-BötC pre-I neurons as part of generating emotional behaviors. We tested this hypothesis by recording single unit activity of the pre-BötC pre-I neurons during stimulation in various regions of the PAG.

## MATERIALS AND METHODS

### Experimental protocols

Experiments were designed and performed at The University of Sydney. Approval for the study was obtained from the institutional Animal Care Ethics Committee. Adult male Sprague-Dawley rats (n = 22) weighing 350–450 g were used for the study. First, the ventrolateral medulla (VLM) just caudal to the facial nucleus was stereotaxically mapped for pre-I cells (coordinates adapted from Paxinos and Watson, 1997). Once a cell had been identified as a pre-I cell by correlating its spike discharge with that of the diaphragm electromyogram (EMG), the cell recording was amplified and displayed on an analog as well as on a digital storage oscilloscope (Scope software; Maclab Systems; AD Instruments Australia) to ensure that the unit under study could unambiguously be discriminated throughout the experiment. The pre-I cell activity signal was also fed into a window discriminator (Labchart software) for continuous assessment of the configuration, shape, and height of the recorded action potentials. After the localization of a pre-I neuron and optimization of its spike discharge, stimulation took place in the dorsal, lateral, and ventrolateral PAG, and changes in the activity of the same pre-I cell were recorded. Each PAG region was stimulated twice to ensure reproducibility of the effect before repositioning the stimulation electrode to another region in the PAG. Once various PAG regions had been stimulated and their effects on a particular pre-I cell examined, the recording electrode in the VLM was moved to track and record another pre-I neuron. After localization of a new pre-I neuron, the same PAG stimulation protocol was applied. In each rat, three or four pre-I cells in the VLM were recorded, and 17–32 microinjections were made in the PAG. To eliminate any pharmacological effect of a previous injection, subsequent microinjections in the PAG were made with an interval of at least 25 minutes.

During the experiments, the cyclic discharge of the nucleus ambiguus (NA), containing pharyngeal and laryngeal motoneurons that also play a role in respiration, might interfere with in vivo localizing of pre-I neurons. The reason is that the NA is situated in the VLM close to the pre-BötC region (Schwarzacher et al., 1995; Guyenet and Wang, 2001; Alheid and McCrimmon, 2008). Because pentobarbitone anesthesia suppresses the spontaneous activity of the NA motoneurons (Merrill, 1970, 1974), as in our previous study (Subramanian, 2013), this anesthesia was used instead of decerebrate unanesthetized preparations.

### Surgery

The rats were initially anesthetized with sodium pentobarbitone (Nembutal, 70 mg/kg, i.p). Catheters were placed in the femoral artery and vein for monitoring blood pressure (BP) and administration of supplementary fluids. To maintain adequate and stable levels of anesthesia, additional doses of Nembutal (5 mg/kg) were delivered intravenously. The depth of anesthesia was assessed by checking the absence of the withdrawal reflex and by measuring arterial blood pressure changes following a hindpaw pinch. A tracheal cannula was inserted via tracheostomy to allow spontaneous breathing but also, if required, to connect a mechanical ventilator. The animal's body temperature was maintained between 36°C and 38°C with a feedback-controlled heating blanket and a rectal probe. Animals were placed in a stereotaxic frame. An occipital craniotomy was performed to allow access to the medulla, and a burr hole was drilled in the skull to allow access to the midbrain PAG. The brain was covered with paraffin oil.

### Diaphragm EMG recording

To measure inspiratory motor output through diaphragm EMG activity, two Teflon-coated stainless-steel wires (0.0045 inch), stripped for 2 mm at each end, were surgically implanted into the crural diaphragm. In all rats, the same part of the muscle was gauged via visual examination in order to implant the EMG electrodes each time in the same part of the muscle, so that similar electrode signal output was obtained. Electrode placements were verified at the conclusion of each experiment.

### Extracellular recording of pre-BötC pre-I neurons

To localize the pre-I neurons in the rostral ventrolateral medulla, micropipettes in a double-barrel cluster were used. The micropipette for cell recording was filled with 3 M NaCl (DC impedance ∼8–10 MΩ). The other micropipette was filled with D,L,homocysteic acid (DLH; 50 mM). The moment when a pre-I cell was detected in the ventrolateral medulla, a 3–6-nl DLH solution was injected at the recording site via the NaCl barrel. This DLH stimulation checked whether the pre-I cell showed excitation as a result of the DLH administration to ensure that the recording was made from the cell body and not from fibers of passage (Fries and Zieglgansberger, 1974; Goodchild et al., 1982; Lipski et al., 1988). For staining the recording area, in eight of a total of 22 animals, rhodamine-B microspheres, added to the DLH electrode, were used to stain the pre-I neuron recording sites. The location of these rhodamine microspheres was assessed later through fluorescence microscopy.

### EAA microinjections into the PAG

For stimulation in the PAG, single-barrel micropipettes (tip diameters 10–30 μm; filled with 50 mM DLH) were inserted into the dorsal, lateral, and ventrolateral PAG. A pressure system (Picospritzer II; Parker Instrumentation) delivered the microinjections. The injected volume was determined using a precalibrated scale and verifying the movement of the meniscus on the scale. Isotonic saline injections were used as controls. In eight animals, rhodamine-B microspheres were added to the DLH solution to determine later the location of the injection sites.

### Histology

At the end of each experiment, the animal was deeply anesthetized and transcardially perfused with 0.9% saline, followed by 4% paraformaldehyde in phosphate buffer, pH 7.2. After perfusion, the brain was removed and stored in 4% formaldehyde for 2 hours, after which it was transferred for at least 48 hours into a 30% sucrose/formaldehyde mixture to prevent formation of ice crystals. The midbrain and brainstem were cut on a freezing microtome into 50-μm coronal sections. The depth of each injection site was noted from the stereotaxic micromanipulator record. When the injection sites were marked by rhodamine microspheres, they were identified by using fluorescence microscopy. Digital images of these sections were captured with an Olympus microscope equipped for epifluorescence and a coupled device connected to a Quickcapture frame grabber board and an Apple Macintosh computer running Adobe Illustrator software CS3. All injection sites were represented on standard drawings according to the stereotaxic atlas of Paxinos and Watson (1997).

### Data analyses

The PCM Vetter-Maclab-Macintosh (SDR Scientific, Sydney, Australia; ADI Instruments, Sydney, Australia) data acquisition system was used for collecting data. In MacLab software (AD Instruments), ensemble averages were derived from amplified (×2,000), bandpass-filtered (20–1,000 Hz) signals using sampling rates of 20,000/second. The pre-I neuron discharge rate (spikes/second) was measured for a period of 10 breaths during both pre-PAG stimulation (control) and post-PAG stimulation (effect). The latency of the response was defined as the time between the moment of injection into the PAG and the onset of a significant increase in the discharge rate of the pre-I cell (mean ± SE). The duration of the response was defined as the time between the moment of onset of the significantly increased discharge rate of the pre-I cell and the moment when the discharge rate of the pre-I cell returned to pre-PAG injection control values. The peak frequency spread (peak firing rate) of pre-I cells was measured before stimulating the PAG.

The raw diaphragm EMG signal was used for measurement of inspiratory (Ti) and expiratory (Te) durations and respiratory frequency (RF). The duration between the onset of the diaphragm signal and its offset was defined as Ti. The duration between the offset of the diaphragm and its subsequent onset was defined as Te (see Subramanian et al., 2008, Fig. 1; Subramanian and Holstege, 2011; Subramanian, 2013).

**Figure 1:**
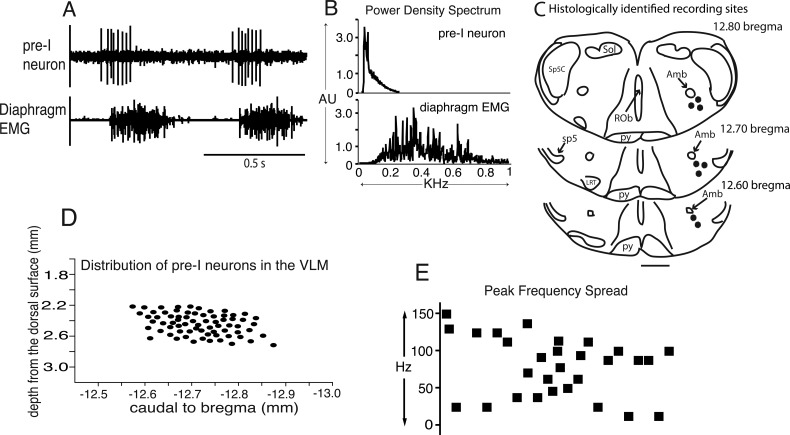
A: Extracellular recording of a pre-BötC pre-I neuron during eupnea, combined with diaphragm EMG. **B:** Power density spectrum of the pre-I neuron during eupnea (computed from n = 10). **C:** Coronal sections of the medulla (adapted from Paxinos and Watson, 1997) showing histologically identified extracellular recording sites (eight cells marked by rhodamine in three rats) from the pre-BötC region. **D:** Spatial distribution of 72 recorded pre-I neurons in the ventrolateral medulla from rostral (−12.9 mm caudal to bregma) to caudal (−12.55 mm caudal to bregma). This zone corresponds to the pre-BötC region. The figure represents a composite of 22 experiments. **E:** Peak frequency discharge spread computed from 29 pre-I neurons.

Statistical comparisons for RF, Ti and Te, and DLH effects were carried out by ANOVA. Statistical comparisons between control and PAG-induced modulation of pre-I cells utilized two-tailed, paired *t*-tests. Computer chart records were transferred into a drawing program (Adobe Illustrator) for data presentation.

### Power density spectral analysis

The spectrum module of the chart software was used to plot the power density spectra of the diaphragm EMG and that of the pre-I cells. The power density spectrum uses a discrete fast Fourier transform (FFT) algorithm (Chart software; AD Instruments) to convert data from time to frequency domains. We selected a FFT size of 1 kHz for representation of the spectrum in the frequency domain. For each signal, the power density spectral windows were computed for 10 successive breaths. All individual power spectral windows were averaged, and the spectra (FFTs) were presented as connected points. The X-axis represents a frequency spread from 0 to 1 kHz, and the Y-axis represents arbitrary units (AU).

## RESULTS

Spontaneously breathing, vagi-intact, pentobarbitone-anesthetized adult rats (n = 22) had the following respiratory and cardiovascular parameters (control values expressed as mean ± SE): inspiration time (Ti) 0.30 ± 0.05 seconds, expiration time (Te) 0.50 ± 0.10 seconds, respiratory frequency (RF) 75 ± 10 breaths/minute, blood pressure (BP) 100 ± 5 mm Hg, heart rate (HR) 380 ± 5 beats/minute, arterial blood pH 7.4 ± 0.01, PaCO_2_ 35.2 ± 3.0 Torr, and PaO_2_ 120.5 ± 5.0 Torr.

### Preinspiratory neurons

During eupnea, the pre-I neurons (n = 72) started firing 15–20 msec prior to the onset of the diaphragm EMG, with a maximum spike frequency during the switch from expiration to inspiration, followed by a declining discharge pattern during early inspiration (Fig. 1A). Their peak power density occurred just before or at the onset of the diaphragm EMG (Fig. 1B). Within the ventrolateral medulla, the dense cluster of pre-I cells was found in a region 12.60–12.84 mm caudal to bregma, 2–2.5 mm lateral to the midline, and 2–3 mm below the dorsal surface. This region was located ventral and ventrolateral to the nucleus ambiguus (NA) and corresponds to the pre-BötC complex region in the adult in vivo rat. Figure 1C illustrates coronal sections of the medulla, showing histologically identified extracellular recording sites (eight cells marked by rhodamine) from the pre-BötC region. Figure 1D illustrates the spatial distribution of the 72 studied pre-I neurons in the ventrolateral medulla. Quantitative analysis of 29 of these 72 pre-I neurons demonstrates that their peak frequency (peak firing rate) ranged from 10 to 150 Hz (Fig. 1E) during eupnea.

### Effects of DLH stimulation of pre-I neurons in the ventrolateral medulla

**Figure 2:**
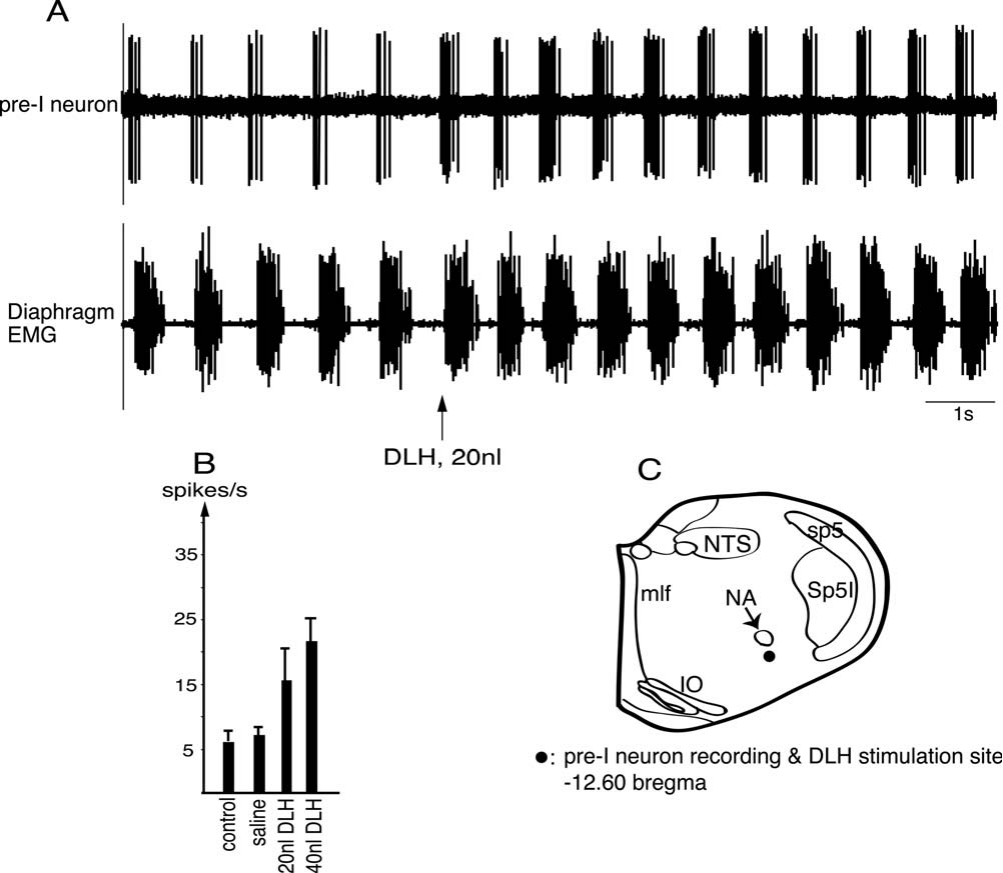
A: Phasic activation of a pre-I neuron following DLH stimulation of the recorded area in the pre-BötC region. **B:** Histogram illustrating dose-dependent phasic excitation of pre-I neurons (n = 10). **C:** Pre-I neuron recording site.

After stimulation (n = 10; DLH, 20 nl) of the pre-I neurons while recording their activity simultaneously (Fig. 2A), the Ti increased from 0.30 ± 0.05 seconds to 0.45 ± 0.05 seconds (*P* < 0.05), and the Te decreased from 0.50 ± 0.15 seconds to 0.25 ± 0.05 seconds (*P* < 0.05), evoking an increase in the RF from 75 ± 5 breaths/minute to 110 ± 10 breaths/minute (*P* < 0.05). The stimulation resulted in an increase in firing of the pre-I cells (Fig. 2A; n = 10) from 7 ± 2 spikes/second (control) to 15 ± 6 spikes/second (*P* < 0.01) for a 20-nl DLH microinjection and to 22 ± 4 spikes/second (*P* < 0.01) for a 40-nl DLH microinjection (Fig. 2B). The pre-I neurons maintained their phase relationship with the crural diaphragm discharge during the stimulation. Figure 2C illustrates the recording and DLH stimulation site of the pre-I neuron illustrated in Figure 2A.

### Effects of DLH stimulation in the PAG on pre-I neuronal activity

**Figure 3:**
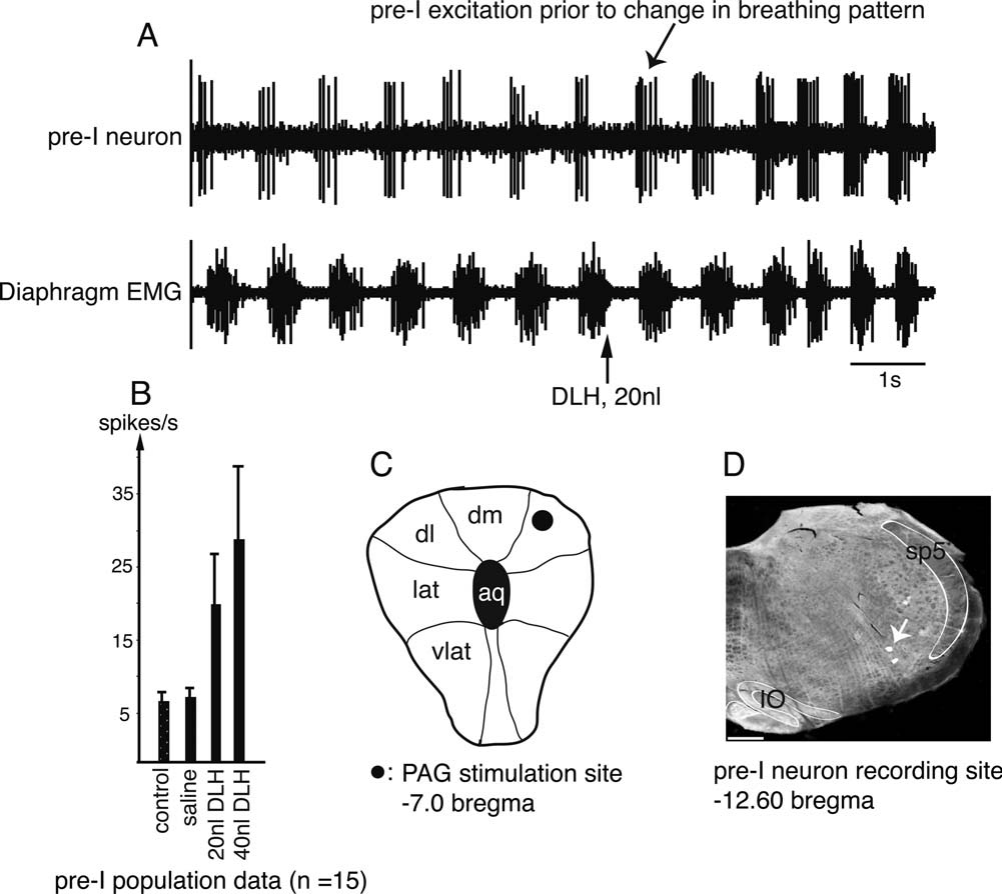
A: Phasic activation of a pre-I neuron following stimulation in the dorsal PAG. **B:** Histogram illustrating dose-dependent phasic excitation of pre-I neurons (n = 15). A 40-nl DLH dose induced a greater increase in spiking of the pre-I cells than a 20-nl DLH dose. **C:** PAG stimulation site. **D:** Pre-I neuron recording site. aq, Midbrain cerebral aqueduct; dm, dorsomedial; dl, dorsolateral; lat, lateral; vlat, ventrolateral, NA, nucleus ambiguus; sp5I, spinal trigeminal nucleus pars interpolaris; sp5, spinal trigeminal tract; NTS, nucleus tractus solitarius; MLF, medial longitudinal fasciculus; IO, inferior olive. Scale bar = 0.5 mm.

**Figure 4:**
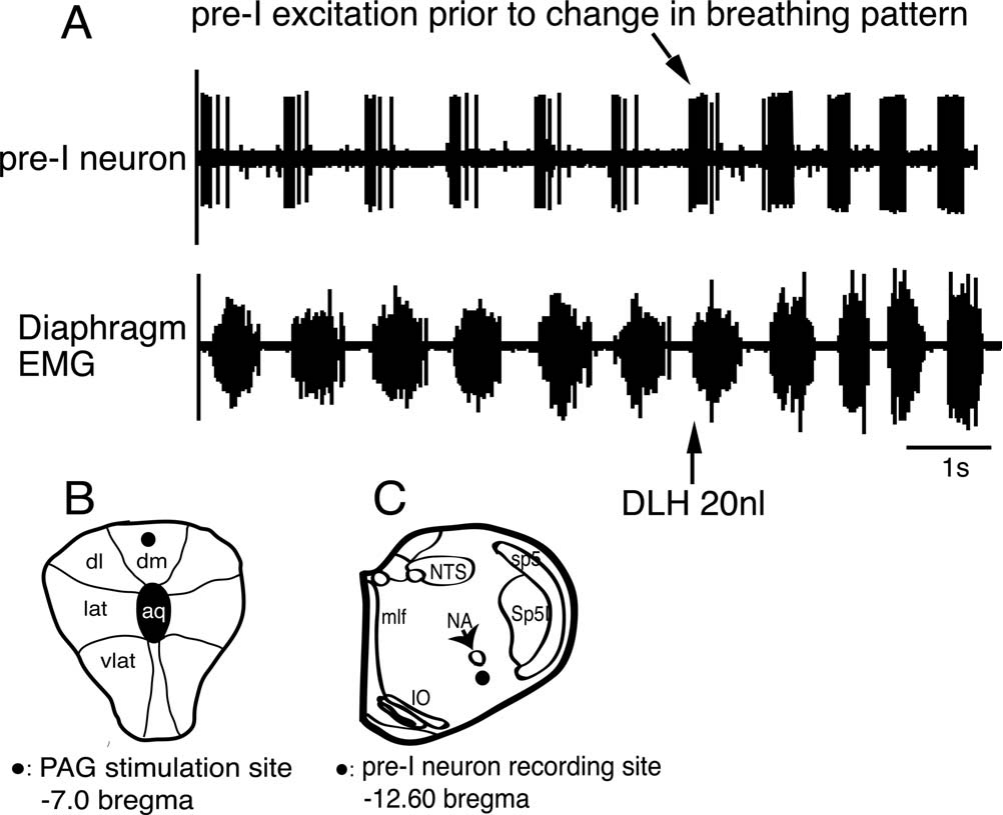
A: Phasic activation of a pre-I neuron following stimulation in the dorsomedial PAG in a different rat. **B:** PAG stimulation site. **C:** Pre-I neuron recording site. For abbreviations see Figure 3.

**Figure 5:**
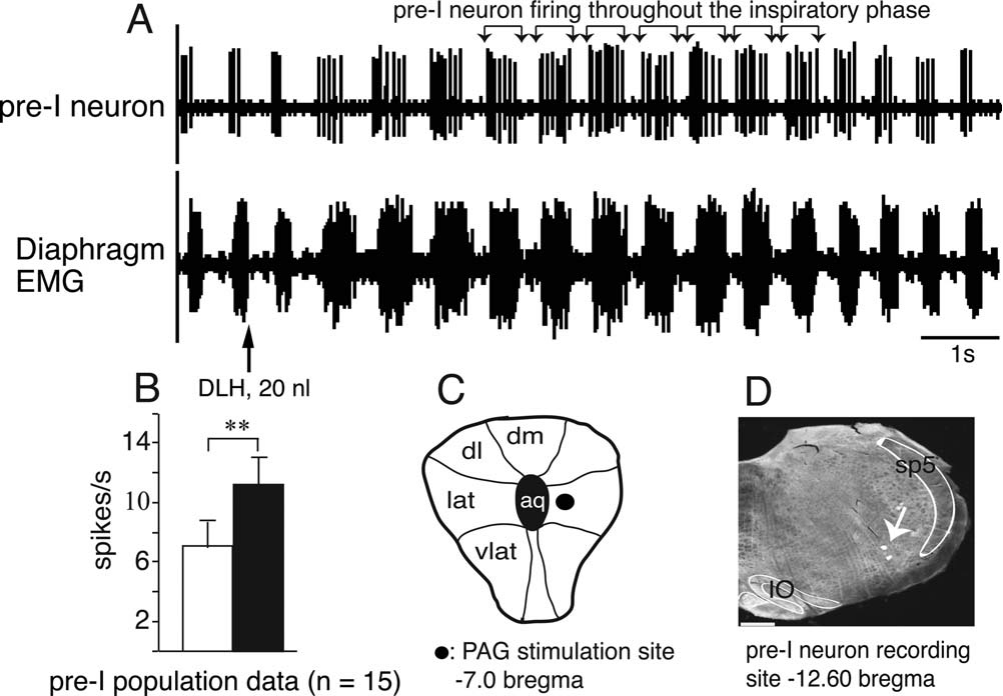
A: Stimulation in the medial part of the lateral PAG (bregma −7.0 mm) converted the pre-I cell into an inspiratory phase-spanning neuron. The pre-I cell started firing 15–30 msec before the diaphragm, as in eupnea, but continued firing throughout inspiration and did not show postinspiratory bursting. **B:** Quantitative changes to pre-I neuronal spike discharge following stimulation in the dorsal part of the lateral PAG (n = 15). **C:** PAG stimulation site. **D:** Pre-I neuron recording site. For abbreviations see Figure 3. Scale bar = 0.5 mm.

**Figure 6:**
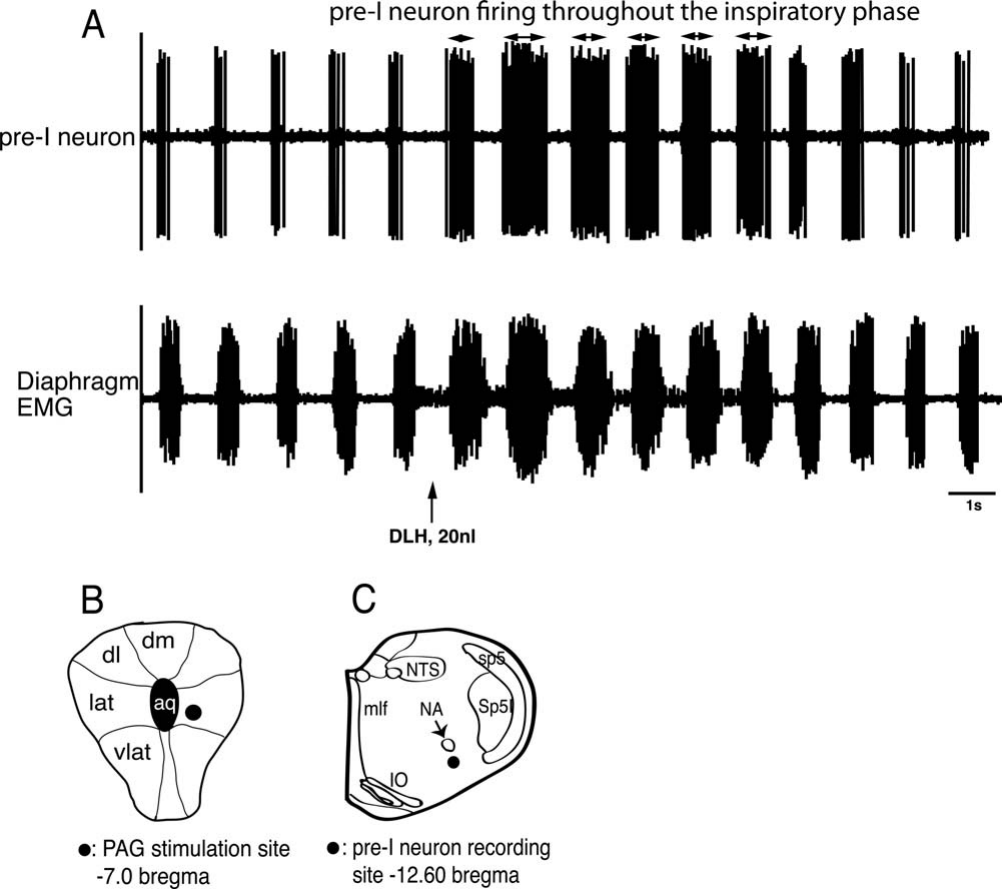
A: Stimulation in the medial part of the lateral PAG (bregma −7.0 mm) in a rat different from that represented in Figure 5 also converted the pre-I cell into an inspiratory phase-spanning neuron. **B:** PAG stimulation site. **C:** Pre-I neuron recording site. For abbreviations see Figure 3.

**Figure 7:**
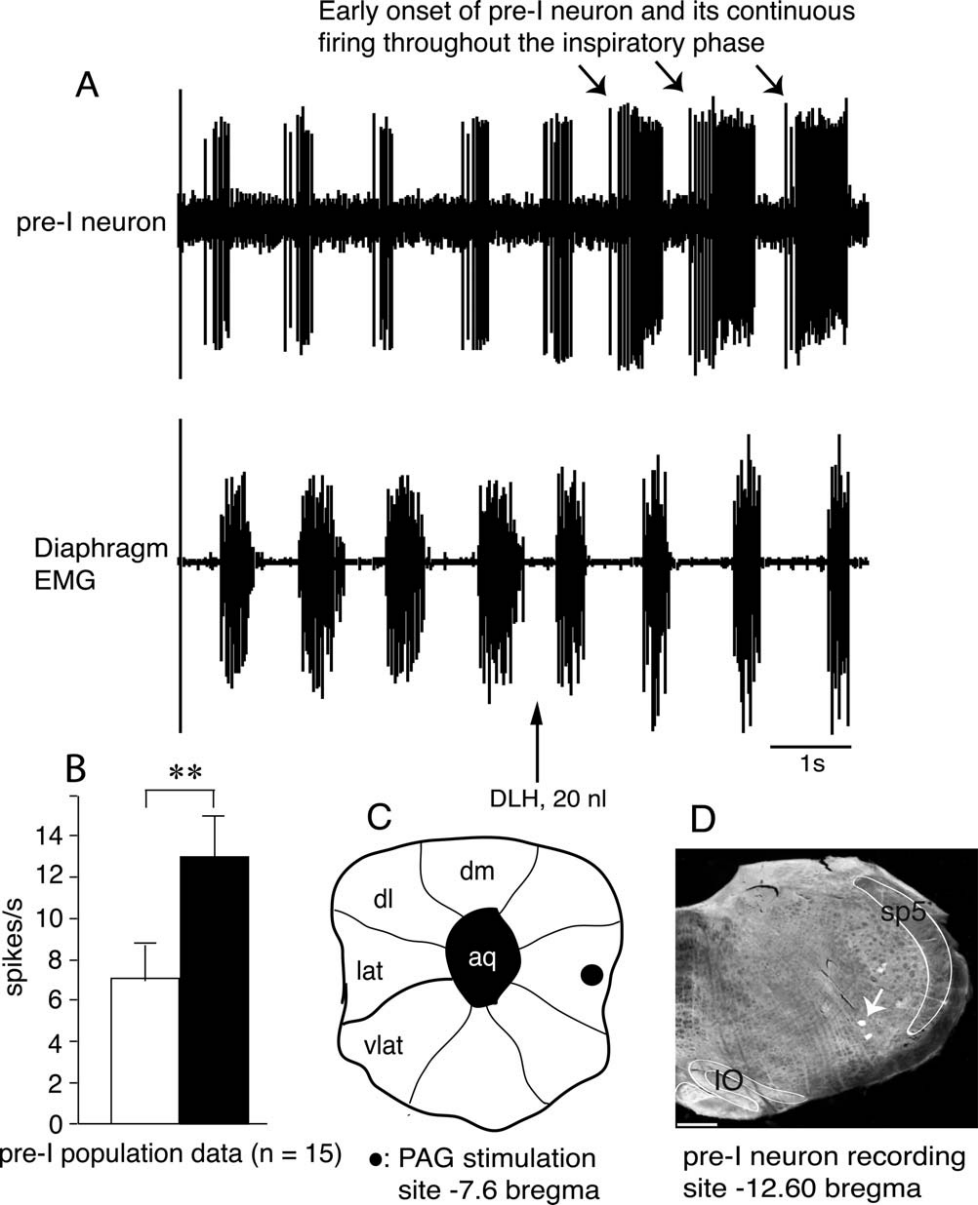
A: Stimulation in the lateral part of the lateral PAG (bregma −7.6 mm) induced an early onset of preinspiratory neuronal discharge. The pre-I neuron started firing 200–400 msec prior to the onset of the diaphragm and continued to discharge throughout the inspiratory phase. **B:** Quantitative changes to pre-I neuronal spike discharge following lateral PAG stimulation (n = 15). **C:** PAG stimulation site. **D:** Pre-I neuron recording site. For abbreviations see Figure 3. Scale bar = 0.5 mm.

**Figure 8:**
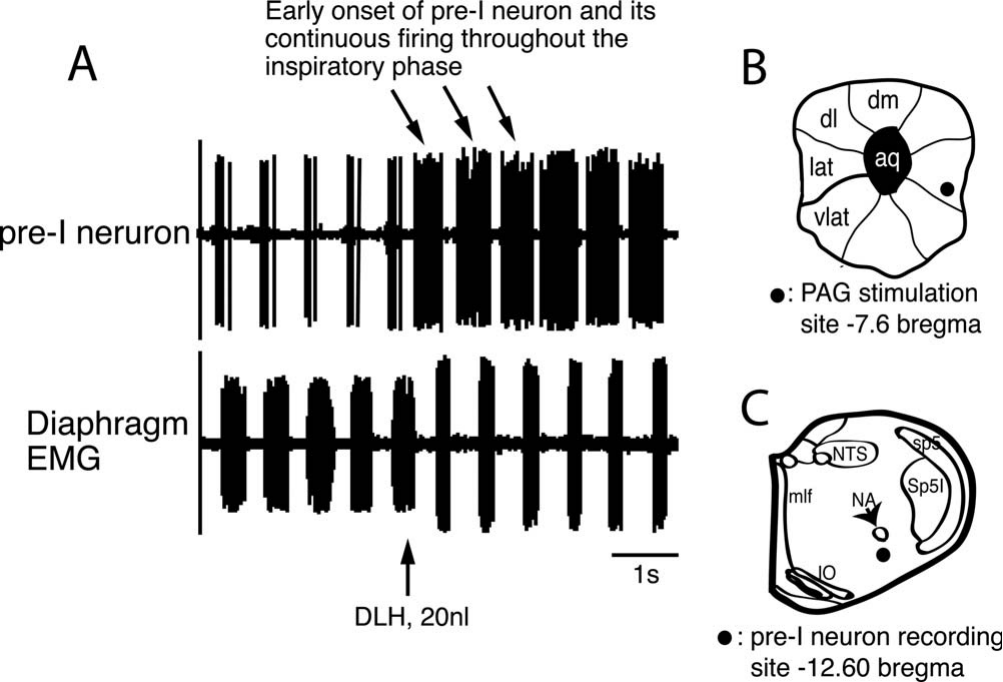
A: Stimulation in the lateral part of the lateral PAG (bregma −7.6 mm) in a rat different from that represented in Figure 7 also induced an early onset of preinspiratory neuronal discharge. **B:** PAG stimulation site. **C:** Pre-I neuron recording site. For abbreviations see Figure 3.

**Figure 9:**
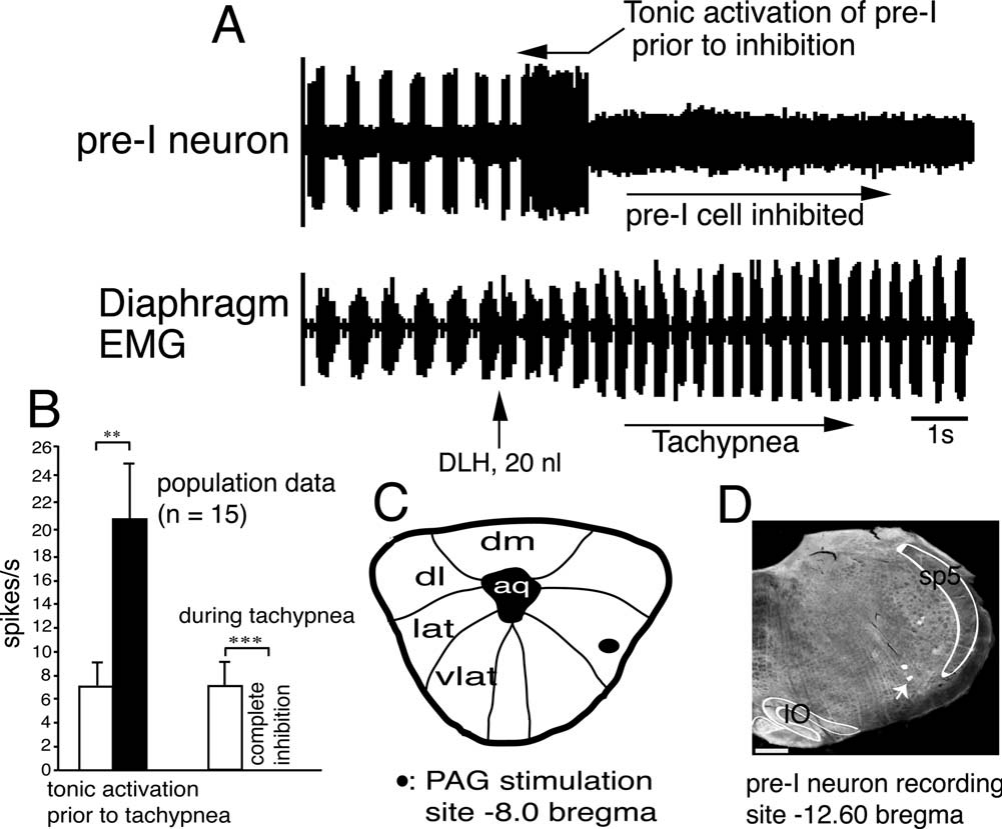
A: Stimulation in the ventral part of the lateral PAG (bregma −8.0 mm) induced tachypnea and tonic activation of the pre-I cell over 1 second, followed by inhibition of the same pre-I cell. **B:** Quantitative changes to pre-I neuronal spike discharge prior to tachypnea and during tachypnea (n = 15). **C:** PAG stimulation site. **D:** Pre-I neuron recording site. For abbreviations see Figure 3. Scale bar = 0.5 mm.

**Figure 10:**
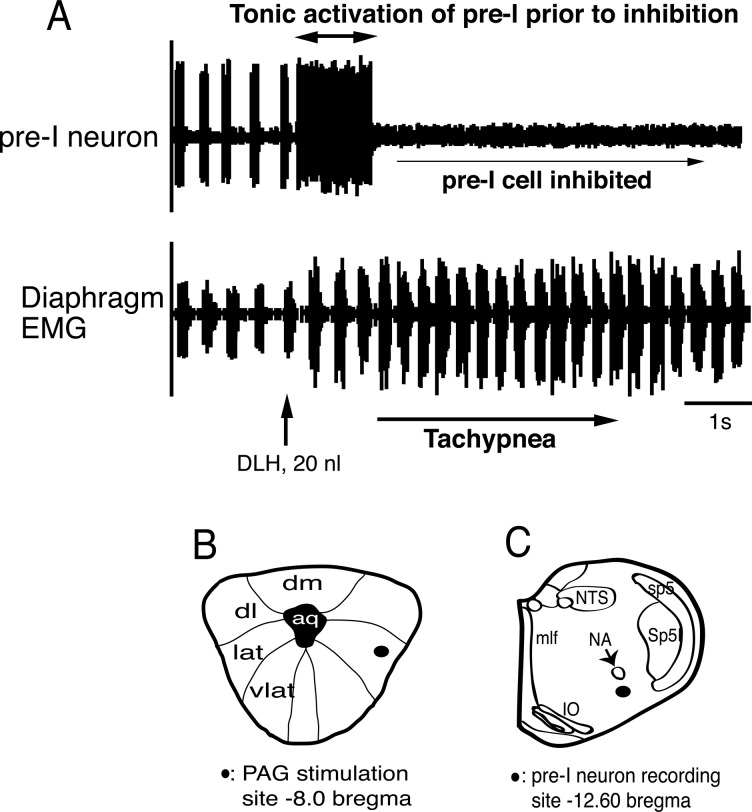
A: Stimulation in a rat different from that represented in Figure 9 in the ventral part of the lateral PAG (bregma −8.0 mm) also induced tachypnea and tonic activation of the pre-I cell over 1 second, followed by inhibition of the same pre-I cell. **B:** PAG stimulation site. **C:** Pre-I neuron recording site. For abbreviations see Figure 3.

**Figure 11:**
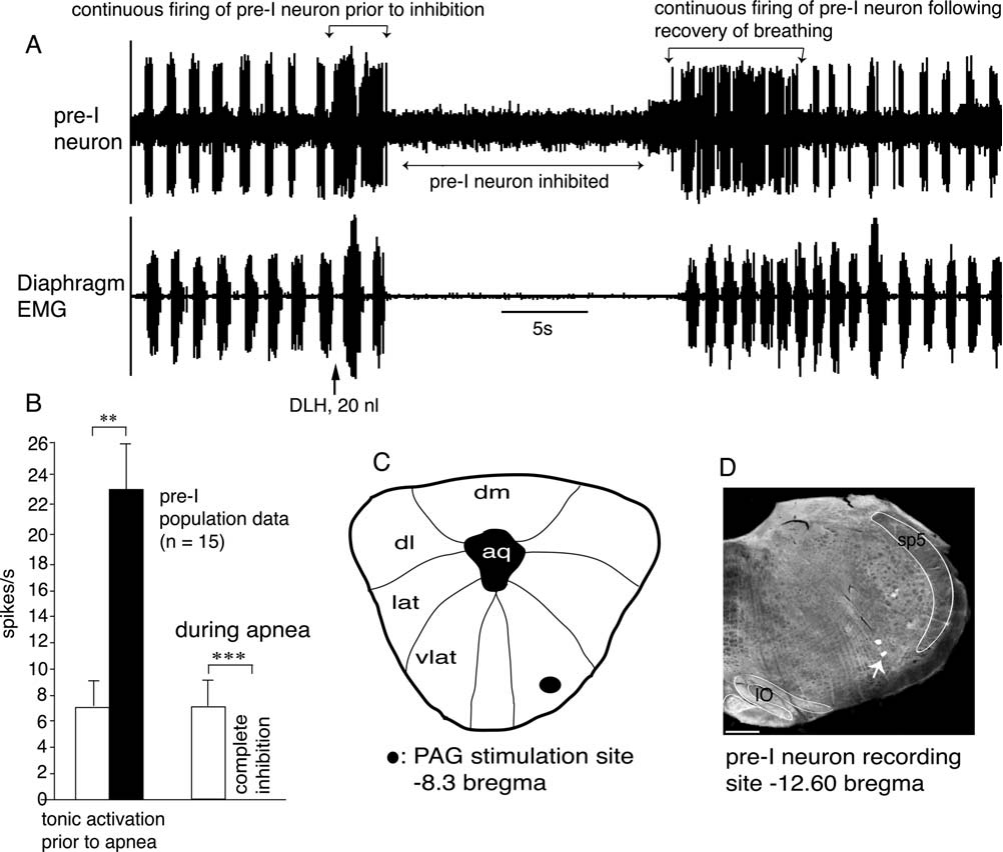
A: Stimulation in the caudal part of the ventrolateral PAG (bregma −8.3 mm) induced apnea and inhibition of the pre-I neuron. Immediately prior to this inhibition, the pre-I cell was tonically activated during two breaths. Following the return of breathing after 17 seconds, the pre-I neuron showed tonic activation during seven breaths before returning to normal eupneic values. **B:** Quantitative changes to pre-I neuronal spike discharge prior to apnea and during apnea (n = 15). **C:** PAG stimulation site. **D:** Pre-I neuron recording site. For abbreviations see Figure 3. Scale bar = 0.5 mm.

Figures 3A, 5A, and 7A illustrate the modulations of the activity of one and the same pre-I cell during stimulation in, respectively, the dorsal PAG, the medial part of the lateral PAG, and the lateral part of the lateral PAG. Figures 9A and 11A illustrate the modulations of another cell in the same rat during stimulation in, respectively, the ventral part of the lateral PAG and the caudal part of the ventrolateral PAG. These data are represented in this way to show that stimulation in different regions of the PAG produces different reactions of the same pre-I cell. The effects represented in Figures 4, 6, 8, and 10 show that stimulation in specific regions in the PAG produces the same effect in the pre-I cells investigated in other rats. Histograms in Figures 3B, 5B, 7B, 9B, and 11B are from analyzing 15 pre-I cells from 15 rats for each effect.

### Dorsal PAG stimulation

Stimulation (n = 20; DLH, 20 nl) in the dorsal (both dorsomedial and dorsolateral) PAG at bregma −7.0 mm and −7.6 mm evoked tachypnea. The Ti decreased from 0.30 ± 0.05 seconds to 0.15 ± 0.05 seconds (*P* < 0.05) and the Te from 0.50 ± 0.15 seconds to 0.25 ± 0.05 seconds (*P* < 0.05), evoking an increase in the respiratory frequency from 75 ± 5 breaths/minute to 140 ± 10 breaths/minute (*P* < 0.05). In Figure 3 the same pre-I neuron is shown as in Figures 5 and 7, whereas in Figure 4 the effect on a different pre-I neuron from a different rat in the same dorsal region is shown. Both neurons (Figs. 3A, 4A) show that dorsal PAG stimulation resulted in a phasic increase in the firing of the pre-I neurons. The pre-I cells (n = 15) increased their discharge rate from 7 ± 2 spikes/second (control) to 19 ± 7 spikes/second (*P* < 0.01; Fig. 3B), a more than 160% increase in their discharge rate compared with preinjection levels, and after a 40-nl DLH injection a more than 300% increase (Fig. 3B). The pre-I neurons maintained their phase relationship with the crural diaphragm discharge during the dorsal PAG induced tachypnea. Figures 3C and 4B illustrate the PAG stimulation site and Figures 3D and 4C the recording site of the pre-I neuron represented in Figures 3A and 4A.

### Lateral PAG stimulation

#### Medial part of the lateral PAG

Stimulation (n = 15; DLH, 20 nl) in the medial part of the lateral PAG (−7.00 mm bregma) generated an increase in Ti from 0.30 ± 0.05 seconds to 0.50 ± 0.10 seconds (*P* < 0.05) and a decrease of Te from 0.50 ± 0.15 seconds to 0.30 ± 0.05 seconds (*P* < 0.05), leaving the RF unchanged at 75 ± 5 breaths/minute. In Figure 5 the same pre-I neuron is shown as in Figures 3 and 7, and in Figure 6 the effect on a different pre-I neuron from a different rat is shown. Both neurons show that stimulation in the medial part of the lateral PAG converted the pre-I neurons into inspiratory phase-spanning cells (Figs. 5A, 6A), because the cells continued firing throughout inspiration. The neurons ceased their activity at the same time as the diaphragm EMG and did not show postinspiratory bursting. During this phase-spanning effect, the pre-I cells (n = 15) firing increased from 7 ± 2 spikes/second to 11 ± 2 spikes/second (*P* < 0.01; Fig. 5B). Figures 5C and 6B illustrate the PAG stimulation site and Figures 5D and 6C the recording site of the pre-I neurons represented in Figures 5A and 6A respectively.

#### Lateral part of the lateral PAG

Stimulation in the lateral part of the lateral PAG (n = 15, bregma −7.6 mm) decreased Ti from 0.30 ± 0.05 seconds to 0.15 ± 0.05 seconds (*P* < 0.05), and Te increased from 0.50 ± 0.15 seconds to 0.75 ± 0.15 seconds (*P* < 0.05), leading to a decrease in the RF from 75 ± 5 breaths/minute to 60 ± 5 breaths/minute (*P* < 0.05). In Figure 7 exactly the same pre-I neuron is shown as in Figures 3 and 5, and in Figure 8 the effect on a different pre-I neuron from a different rat is shown. Both neurons show that DLH stimulation in the lateral part of the lateral PAG induced an earlier onset of preinspiratory discharge, 200–400 msec prior to the onset of the diaphragm (Figs. 7A, 8A), which continued throughout the inspiratory phase. The offset times of the cells were time-locked with the offset of the diaphragm activity. During this inspiratory phase-spanning effect, the pre-I cells (n = 15) firing frequency increased from 7 ± 2 spikes/second to 13 ± 2 spikes/second (*P* < 0.01; Fig. 7B). Figures 7C and 8B illustrate the PAG stimulation site and Figure 7D and 8C the recording site of the pre-I neurons represented in Figures 7A and 8A, respectively.

#### Ventral part of the lateral PAG

DLH stimulation in the ventral part of the lateral PAG (n = 20, bregma −8.0) resulted in tachypnea. Ti decreased from 0.30 ± 0.05 seconds to 0.15 ± 0.05 seconds (*P* < 0.05), and Te decreased from 0.50 ± 0.15 seconds to 0.25 ± 0.05 seconds (*P* < 0.05), resulting in an increase in RF from 75 ± 5 breaths/minute to 150 ± 10 breaths/minute (*P* < 0.05). In Figure 9 the same pre-I neuron is shown as in Figure 11, and in Figure 10 the effect on a different pre-I neuron from a different rat is shown. Both neurons represented in Figures 9 and 10 show that DLH stimulation in the ventral part of the lateral PAG generated a tonic activation of the pre-I cells (n = 15) of over 1 second (Fig. 9A, 10A), with a peak discharge of 21 ± 4 spikes/second (*P* < 0.01; Fig. 9B). After this period of tonic activation, the pre-I neurons abruptly stopped firing (Figs. 9A, 10A), which lasted for more than 25 seconds before the cells reactivated. Figures 9C and 10B illustrate the PAG stimulation site, and Figures 9D and 10C show the recording site of the pre-I neurons represented in Figures 9A and 10A, respectively.

### Ventrolateral PAG stimulation

**Figure 12:**
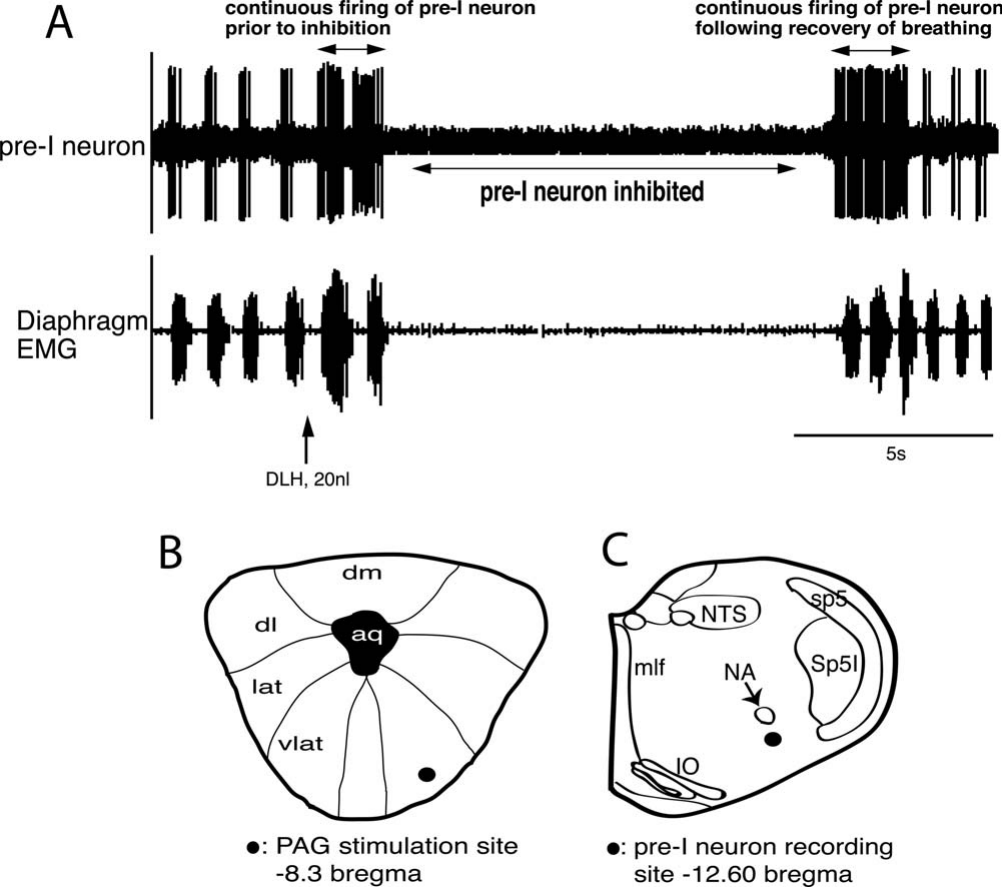
A: Stimulation in the caudal part of the ventrolateral PAG (bregma −8.3 mm) in a rat different from that represented in Figure 11 also induced apnea and inhibition of the pre-I neuron. Immediately prior to this inhibition, the pre-I cell was tonically activated during two breaths. Following the return of breathing after 16 seconds, the pre-I neuron showed tonic activation during three breaths before returning to normal eupneic values. **B:** PAG stimulation site. **C:** Pre-I neuron recording site. For abbreviations see Figure 3.

DLH stimulation in the caudal part of the ventrolateral PAG (n = 15, bregma −8.3 mm) resulted in a tonic activation of the pre-I cells (Figs. 1A, 2A), exhibiting a discharge of 23 ± 3 spikes/second (n = 15, *P* < 0.01; Fig. 11B). In Figure 11 the same pre-I neuron is shown as in Figure 9, and in Figure 12 the effect on a different pre-I neuron from a different rat is shown. After this activation period, the pre-I neurons stopped firing as well as the diaphragm muscle for about 20 seconds. When the pre-I neurons restarted firing, the diaphragm also reactivated. After the period of apnea, the pre-I neurons were tonically activated during several breaths before returning to prestimulation values, accompanied by an increased RF of 130 ± 10 breaths/minute (*P* < 0.05) for 5–10 seconds. Figures 1C and 2B illustrates the PAG stimulation site and Figures 1D and 2C the recording site of the pre-I neurons represented in Figures 1A and 2A, respectively.

## DISCUSSION

A high density of pre-I cells was found in the pre-BötC region just ventral to the nucleus ambiguus, 12.55–12.85 mm caudal to bregma or 0.6–0.8 mm rostral to the obex. Directly outside this pre-I neuron region, a diversity of other inspiratory- and expiratory-related neurons was recorded. Our results show that dorsal PAG stimulation generates not only an increase in RF but also larger inspiratory efforts. The dorsal PAG modulation of the pre-I neurons had no impact on their rhythmogenic property, because they remain time-locked with the diaphragm. Stimulation in the medial part of the lateral PAG converted the pre-I cells into inspiratory phase-spanning cells, resulting in inspiratory apneusis, whereas stimulation in the lateral part of the lateral PAG induced an earlier onset of preinspiratory neuronal discharge and shortening of the inspiration time. DLH stimulation in the ventral part of the lateral PAG induced tachypnea. Although prior to the start of this tachypnea the pre-I neurons were tonically activated, they were subsequently inhibited, which means that the pre-I cells did not play a role in generating this tachypnea. Stimulation in the ventrolateral PAG inhibited not only the pre-I cells but also the diaphragm.

### Pre-BötC region in vivo

Most neurophysiological studies verify the location of the extracellular recording site in the pre-BötC by documenting the stereotaxic location noted from the stereotaxic micromanipulator. In most studies in which the cell recording site is shown by means of staining, the recording electrode is first removed, followed by inserting a new staining electrode into the same area. Obviously, this technique does not accurately localize the exact site of recording because of the electrode removal and reinsertion. For this reason, in eight of 22 animals, rhodamine-B microspheres were added to the DLH electrode, to stain the pre-I neuron recording sites immediately after recording these cells. In in vivo animals, the boundaries of the pre-BötC region in the ventrolateral medulla are vague. For the in vivo cat, Dobbins and Feldman (1994) and Schwarzacher et al. (1995) used the obex coordinates as stereotaxic references for electrophysiological isolation of pre-I neurons in the pre-BötC region. The dense cluster of pre-I cells was found just ventral to the nucleus ambiguus. A similar location was found in the in vivo rat (Sun et al., 1998; Guyenet and Wang, 2001; Monnier et al., 2003; Tan et al., 2010) as well as in the in situ rat (Paton et al., 2006; St. John, 2009) and in the in situ mouse (Paton, 1996). All these studies suggested that, within the ventrolateral medulla, the pre-BötC region is the main location of the pre-I interneurons. In the present study in the rat, the rostral ventrolateral medulla was systematically mapped between 0 to 1.5 mm rostral to the obex. A high density of pre-I cells was found just ventral to the nucleus ambiguus, 12.55–12.85 mm caudal to bregma or 0.6–0.8 mm rostral to the obex. Directly outside this pre-I neuron region, a diversity of other inspiratory- and expiratory-related neurons was recorded. On the basis of the high density of pre-I cells and anatomical reconstruction just ventral to the nucleus ambiguus, we classified this area as representing the pre-BötC region in vivo. Although in much lower numbers than the pre-I cells, other types of respiratory neurons were also found in this region, such as preinspiratory–inspiratory (pre-I/I) neurons, inspiratory-decrementing neurons, and postinspiratory cells (Subramanian and Holstege, 2011; Subramanian, 2013).

In previous studies (Schwarzacher et al., 1995; Guyenet and Wang, 2001) the pre-I neuron classification was made by examining cell activity in correlation with the phrenic nerve discharge. We used the diaphragm EMG instead, which is a standard practice for measuring central respiratory rhythm. In fact, the time difference between the phrenic nerve discharge and the contraction of the diaphragm is less than 5 msec. Because the pre-I cells start 15–30 msec prior to the onset of the diaphragm, this approach enabled us to isolate and classify the pre-I neuron unequivocally. The spiking onset of the pre-I cells coincided with the onset of stage 2 of the expiratory phase as defined by Schwarzacher et al. (1995). From this stage onward, the spike discharge of the pre-I neurons slowly increased and reached its peak activity just before the onset of the diaphragm EMG. The peak frequency spectrum and interspike intervals of the pre-I cells as seen in vivo are similar to those from in-situ-perfused rats and mice (Paton, 1996; Paton et al., 2006; St. John, 2009). We did not verify whether the pre-I neurons possess intrinsic pacemaker mechanisms (Feldman and Del Negro, 2006; Feldman et al., 2012) or whether their rhythmic bursting is network driven (Richter and Spyer, 2001; Doi and Ramirez, 2008; St. John, 2009; Smith et al., 2009, 2012) but investigated whether pre-I cells can be modulated by neurons in the PAG.

### Difference between stimulation of pre-BötC region and stimulation of PAG regions for pre-I neuron discharge and diaphragm function

DLH stimulation of recorded pre-I cells in the pre-BötC region increased their discharge rate, increased the inspiratory duration, and decreased the expiratory duration, leading to an increased RF. The amplitude of the diaphragm EMG remained unchanged. The increase in RF following DLH injection and the pattern of pre-I cell activity are consistent with the pre-I cells in the pre-BötC as defined by Smith et al. (1991) in vitro and Monnier et al. (2003) in vivo and may represent state-dependent modulation (Doi and Ramirez, 2008) in vivo. However, in the case of vocalization, vomiting, parturition, etc., the automatic breathing pattern is interrupted. The role of the PAG region in switching respiratory phases, following electrical stimulation has long been known (Hockman et al., 1974). The PAG controls these activities by modulating the medullary premotor interneurons involved in them, in particular the late-I and post-I neurons (Subramanian, 2013), but also by changing pre-I neuron activity, generating a different respiratory rhythm and diaphragm patterning.

### Effect of the dorsal PAG on pre-I neurons

In humans, stimulation in the dorsal PAG evokes elimination of pain (Nashold et al., 1969, see Linnman et al., 2012), unpleasant sensations such as burning and cold, intense distress, bladder voiding urge, sweating, panic, terror, and feelings of imminent death (Hosobuchi, 1987; Del-Ben and Graeff, 2009) as well as an increase in peak expiratory flow rate (Hyam et al., 2012). In the rat, stimulation in the dorsal PAG generates avoidance and aversion behavior, micturition, defecation, and escape responses (Del-Ben and Graeff, 2009). The behaviors described above are accompanied by increases in inspiratory effort as well as tachypnea and hyperventilation (Huang et al., 2000; Hayward et al., 2004; Zhang et al., 2007; Subramanian, 2013) and vasomotor responses (Horiuchi et al., 2009; Iigaya et al., 2010). Our results show that dorsal PAG stimulation induced an increase in the discharge of the pre-I neurons, leading to an increase in inspiratory effort. Dorsal PAG stimulation had no impact on the rhythmogenic property of the pre-I neurons, because dorsal PAG stimulation did not disrupt the time lock between pre-I neurons and diaphragm function. Subramanian (2013) showed that stimulation in the dorsal PAG also generated an increase in the discharge of the late-I and post-I neurons in the ventrolateral medulla. The late-I and post-I neurons are thought to mediate the inspiration-to-expiration transition, i.e., to function as the inspiratory Off switch (Richter, 1982; Richter et al., 1987; Smith et al., 2009; Dutschmann et al., 2009). Thus the increase in the phasic discharge of all the three cells, the pre-I, late-I, and post-I, may contribute to shaping different components of tachypnea, such as inspiratory effort, inspiratory and expiratory durations, and RF depending on the behavior generated.

### Effect of lateral PAG stimulation on pre-I neurons

In humans, electrical stimulation in the dorsal portion of the lateral PAG evokes feelings of choking and sensations of fear, whereas stimulation in the lateral and ventral portions of the lateral PAG evokes strong emotional reactions, including aggression (Nashold et al., 1969). In cats, stimulation in both the lateral and the ventral part of the lateral PAG generates aggressive (fight) or escape (flight) behavior, including vocalization (Bandler, 1971; Bandler et al., 1972; Bandler and Carrive, 1988; Bandler and Depaulis, 1991; Carrive, 1993; Zhang et al., 1994; Subramanian et al., 2008). Stimulation in the lateral PAG in the rat has also been shown to unleash panic attacks (Schimitel et al., 2012). Subramanian et al. (2008) described the PAG-generated respiratory responses during vocalization, but the respiratory responses during strong emotional reactions as aggression or fear and panic are not known. Most of these behaviors would involve hyperresponsiveness to hypercapnea (Schimitel et al., 2012) generated during these behaviors, which would require modulation of inspiratory duration. This study shows that DLH stimulation in the medial and lateral parts of the lateral PAG transformed pre-I cells into inspiratory phase-spanning cells, resulting in inspiratory apneusis or firing the pre-I cells in the stage 1 instead of stage 2 expiratory phase together with an increase in the late-I neuron discharge and inhibition of the post-I cell discharge (Subramanian, 2013).

Stimulation in the ventral part of the lateral PAG is known to produce fight and flight (Carrive, 1993), in the context of which it generates tachypnea. The present study demonstrates that this tachypnea involves a twofold increase in diaphragm amplitude. Prior to the start of this tachypnea the pre-I neurons were tonically activated, but subsequently inhibited, which means that the pre-I cells did not play a role in generating this tachypnea. Other cells in the ventrolateral medulla, in particular the late-inspiratory (late-I) neurons (Subramanian, 2013) or the nucleus retroambiguus (Holstege, 1989; Subramanian and Holstege, 2009), might play a role in this generation of tachypnea.

### Effect of the ventrolateral PAG on pre-I neurons

In humans, stimulation in the caudal part of the ventrolateral PAG induces feelings of fright and terror (Nashold et al., 1969), and, in conscious cats and rats, it induces freezing and immobility (Zhang et al., 1990; Bandler and Depaulis, 1991; Carrive, 1993). The freezing and immobility reaction prevents the predator from spotting the rat, because most animals, including predators, notice mainly moving objects within their visual fields. When a rat stops all movements, the predator does not detect it, which allows the rat to survive the situation. Were the rat to continue breathing, the predator would observe these movements, resulting in the death of the rat. Thus it is important for the caudal part of the ventrolateral PAG, in the context of the freezing and immobility reaction, to stop all movements, including respiration. This might explain why DLH stimulation in the ventrolateral PAG inhibits both the pre-I cells and the diaphragm. Richter (1982) proposed that, during apnea or breath-holding, the postinspiratory neurons clamp the inspiration-generating mechanisms. This might clarify the continuous activation of the post-I neurons during the caudal ventrolateral PAG-generated apnea (Subramanian, 2013).

### Technical considerations

As in our previous studies (Subramanian and Holstege, 2011; Subramanian, 2013), we used spontaneously breathing rather than artificially ventilated rats. This allowed central respiratory centers to affect breathing normally owing to cyclic changes in chemoreceptor activity resulting from changes in PaO_2_ and PaCO_2_ concentrations. In our study, poikilocapnia was not seen during control conditions. This is reflected in maintenance of stable eupneic respiratory rhythm, stable blood pressure, and stable pre-I cell recordings during eupneic rhythm. This means that the effects immediately following PAG stimulation were PAG induced, although changes in blood gas composition from a PAG-induced effect might have contributed to further shaping the effect. Stimulation of the PAG could also have induced neurohumoral responses (Horiuchi et al., 2009; Iigaya et al., 2010; see Behbehani, 1995), which are similar to sympathetic responses acting on heart and blood vessels, both occurring in the 1–2-second range. Although the duration of a PAG-induced effect was over 1–2 seconds (depending on the DLH dosage), the changes to the pre-I cell discharge following PAG stimulation started within milliseconds, suggestive of a direct relay from the PAG to the pre-I neurons.

## CONCLUSIONS

**Figure 13:**
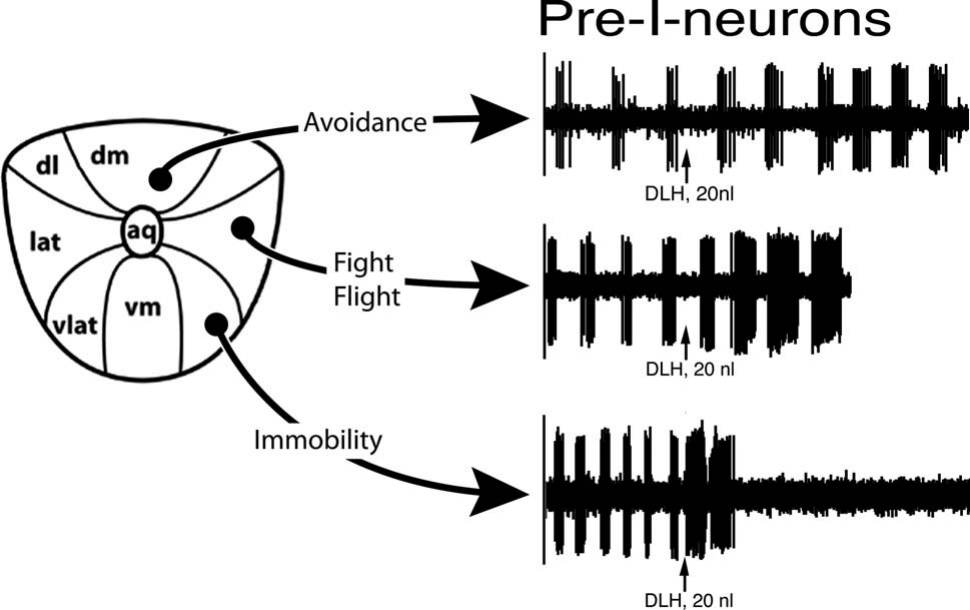
Summary diagram of the effects of PAG stimulation on pre-BötC pre-I neurons. Stimulation in the dorsal PAG produces phasic excitation in the context of avoidance behavior, whereas stimulation in the lateral PAG produces tonic excitation in the context of fight and flight. Stimulation in the ventrolateral PAG produces inhibition of pre-I cells in the context of freezing and immobility.

The PAG is known to generate those behaviors necessary to survive the circumstances in which animals find themselves. The present results show that within this context the PAG also excites pre-I neurons both phasically and tonically, or even silences them, depending on which behavior is necessary (Fig. 13).

## ROLE OF AUTHORS

HHS conceived, designed and performed the experiments and analyzed the data. HHS and GH wrote the paper.
